# Supplementary material for: Influential Factors and Spatiotemporal Characteristics of Carbon Intensity on Industrial Sectors in China
Source: Int J Environ Res Public Health. 2021 Mar 12;18(6):2914. doi: 10.3390/ijerph18062914 (PMC8000731; doi:10.3390/ijerph18062914)
Supplement: Supplementary file 1 [file ijerph-18-02914-s001.pdf]

International Journal of
Environmental Research and Public Health

MDPI

Type of the Paper (Article)

Influential Factors and Spatiotemporal Characteristics of Carbon Intensity on Industrial Sectors in China

Supplementary Materials Table S1: Conversion coefficient and CO_2 emissions conversion coefficient of different kinds of energy.

Table.S1 Conversion coefficient and CO_2 emissions conversion coefficient of different kinds of energy.

	Unit	Coal	Coke	Crude oil	Gasoline	Kerosene	Diesel oil	Fuel oil	Nature gas
NCV_j	（kJ/kg）	20908	28435	41816	43070	43070	42652	41816	38931
C_j	(ton CO_2/TJ)	26.37	29.5	20.1	18.9	19.5	20.2	21.1	15.3
OFj	-	0.94	0.93	0.98	0.98	0.98	0.98	0.98	0.99
f_j	(kgce/kg)	0.714	0.971	1.429	1.471	1.471	1.457	1.429	1.330

Notes: The unit of carbon emission coefficient of natural gas is kgce/m^3

Supplementary Materials Table S2: Codes and names of 20 industrial sectors.

Table.S2 Codes and names of 20 industries.

Industry structure	Industry code	Industry name
Primary industry	S1	Agriculture, Forestry, Fishery, and Fishery industry
Secondary industry	S2	Mining industry
	S3	Food, Beverage and Tobacco Manufacturing
	S4	Textile, Clothing and Leather Products Manufacturing
	S5	Wood Processing and Wood, Bamboo, Cane, palm, and Straw Manufacturing
	S6	Paper Making and Paper Products Manufacturing
	S7	Manufacturing of Oil Processing and Coking Processing
	S8	Manufacturing of Raw Chemical Materials, Chemical Products, and Medicine
	S9	Manufacturing of Rubber and Plastic
	S10	Manufacturing of Non-metal Products
	S11	Smelting and Rolling Process of Ferrous Metal
	S12	Manufacturing of Metal Products
	S13	Manufacturing of Communication Device, Computers, and Other Electronic
	S14	Manufacturing of Electrical Machinery and Equipment
	S15	Manufacturing of Ordinary and Special Machinery

S16	Manufacture of Transport Equipment
S17	Production and Supply of Electricity, Heat, Gas, and Water
S18	Construction industry
S19	Wholesale, Retail, Accommodation, and Catering
S20	Transportation, Storage and Postal Service

Supplementary Materials Table S3: Panel unit root tests

It is necessary to analyze the stability of panel data. The unit root test shows that the panel data of China's industrial carbon intensity from 2005 to 2015 is stable. It has passed the cointegration test, which indicates that there is a long-term stable equilibrium relationship on the variables and the equation regression residuals are stable.

Table.S3 Results of panel unit root tests.

Variables	（c,t,p）	Models	Statistics	Value	P-value
Ln(DCI)	（1,0,1）	Inverse chi-squared(40)	*P*	80.9158	0.0000
		Inverse normal	*Z*	-4.3620	0.0000
		Inverse logit t(104)	*L*	-4.3030	0.0000
		Modified inv. chi-squared	*Pm*	4.5745	0.0000
Ln(WP)	（1,0,1）	Inverse chi-squared(40)	*P*	91.9550	0.0000
		Inverse normal	*Z*	-5.3561	0.0000
		Inverse logit t(104)	*L*	-5.2558	0.0000
		Modified inv. chi-squared	*Pm*	5.8087	0.0000
Ln(IAV)	（1,0,1）	Inverse chi-squared(40)	*P*	132.6944	0.0000
		Inverse normal	*Z*	-6.5581	0.0000
		Inverse logit t(104)	*L*	-7.3380	0.0000
		Modified inv. chi-squared	*Pm*	10.3635	0.0000
Ln(FAI)	（1,0,1）	Inverse chi-squared(40)	*P*	102.3762	0.0000
		Inverse normal	*Z*	-4.6433	0.0000
		Inverse logit t(104)	*L*	-5.0373	0.0000
		Modified inv. chi-squared	*Pm*	6.9739	0.0000
Ln(CR)	（1,0,1）	Inverse chi-squared(40)	*P*	131.9514	0.0000
		Inverse normal	*Z*	-7.8946	0.0000
		Inverse logit t(104)	*L*	-8.0523	0.0000
		Modified inv. chi-squared	*Pm*	10.2805	0.0000
Ln(RTE)	（1,0,1）	Inverse chi-squared(40)	*P*	108.0896	0.0000
		Inverse normal	*Z*	-6.1284	0.0000
		Inverse logit t(104)	*L*	-6.1473	0.0000
		Modified inv. chi-squared	*Pm*	7.6126	0.0000

Note: c=1 means including constant terms, c=0 means not including constant terms, t=1 means including time trend terms, t=0 means not including time trend lines, p=1 means 1 period lagging

Supplementary Materials Table S4: Estimation results of Variance Inflation Factor test.

The results of the Variance Inflation Factor (VIF) test are shown in Table.S4.

Table.S4 Estimation results of VIF test.

Variable	VIF	1/VIF
LnWP	1.89	0.529869
LnIAV	2.48	0.403606
LnFAI	3.03	0.330189
LnCR	1.00	0.996110
LnRTE	2.05	0.488336
Mean VIF	2.12	

Supplementary Materials Table S5: Hausman Test

The value of the Hausman Test for carbon intensity is (53.43,p=0.0000), indicating that the model accepts the null hypothesis of fixed effects. The Hausman test results are shown in the table. It is determined that the space fixed form is used to construct the spatial panel data

Table S5. The result of the Hausman Test.

	(b)	(B)	(b-B)	sqrt(diag(V_b-V_B))
	FE	RE	Difference	S.E.
LnWP	0.1096994	0.0291055	0.080594	0.0329218
LnIAV	-0.9763411	-0.942641	-0.0337001	0.0426614
LnFAI	0.0124609	-0.33127	0.0455879	0.0139446
LnCR	-0.0119748	0.0174571	-0.0054823	0.0015116
LnRTE	0.1095296	0.1550678	-0.0455382	0.0077523
C	6.946525	7.03846	-0.0919351	.
